# Persistent headache burden after surgical treatment of post-dural puncture headache: a cross-sectional study

**DOI:** 10.3389/fneur.2026.1846001

**Published:** 2026-05-25

**Authors:** Ali Kapan, Carolina Iten, Christian T. Ulrich, Frank Donnerstag

**Affiliations:** 1 Center for Public Health, Department of Social and Preventive Medicine, Medical University of Vienna, Vienna, Austria; 2 Department of Neurosurgery, Lindenhofspital, Bern, Switzerland; 3 Institute for Diagnostic and Interventional Neuroradiology, Hannover Medical School, Hannover, Germany

**Keywords:** anesthesia, cerebrospinal fluid leak, epidural, headache disability, labor analgesia, neuraxial, obstetric, post-dural puncture headache

## Abstract

**Background:**

Post-dural puncture headache (PDPH) may follow intended or unintended spinal dural puncture and is usually self-limiting. In some patients, however, symptoms persist despite epidural blood patches, while postoperative patient-reported data are largely absent. We assessed headache disability, functional status, and psychosocial burden among respondents self-reporting prior surgery for PDPH or dural-puncture-related CSF leak.

**Methods:**

An online cross-sectional survey was conducted between February and April 2025. Adults who self-reported prior surgical treatment for PDPH or dural-puncture-related CSF leak were recruited through international online patient support groups. Data were analysed descriptively. Key variables included HIT-6 headache disability, symptom trajectory, occupational status, and psychological symptoms.

**Results:**

A total of 136 respondents were included; 74.3% were female, with a median age of 38 years. Diagnostic or therapeutic lumbar puncture was the most frequent reported cause of CSF leak (65.1%), followed by epidural anesthesia (30.6%). Most respondents had undergone a single surgical intervention (73.1%). Complete symptom remission was reported by 9.6% of respondents. Slight and fluctuating improvement was the most frequent trajectory (38.7%; mean HIT-6 61.5, SD 12.0), followed by significant but persistent improvement (27.1%; mean HIT-6 51.5, SD 6.0); 10.4% reported largely unchanged and 5.1% worsened symptoms.

**Conclusion:**

Among respondents in this self-selected online cohort, persistent headache burden and psychosocial impairment were frequently reported, whereas complete symptom remission was reported by a minority of respondents. Prospective studies with structured follow-up are needed to better characterize postoperative symptom trajectories.

## Background

Postdural puncture headache (PDPH) has long been regarded as a benign, self-limiting complication of dural puncture that resolves in most patients with conservative management or after an epidural blood patch (EBP) (1, 2). However, accumulating evidence suggests that this view is incomplete. A clinically relevant subset of patients develops prolonged or refractory courses characterized by chronic headache following dural puncture, persistent functional impairment, and, in selected cases, the need for invasive or surgical interventions (3, 4). The underlying mechanisms are thought to involve persistent CSF leakage, resulting in intracranial volume depletion and traction on pain-sensitive structures, as well as reflex or compensatory cerebral vasodilation; however, the precise pathophysiology remains incompletely understood (1). Orthostatic headache remains the hallmark feature of PDPH and may be accompanied by neck stiffness, nausea, and visual or auditory disturbances. In prolonged or refractory cases, less typical presentations have also been described, including persistent diffuse or less clearly orthostatic headache, cognitive symptoms (“brain fog”), concentration difficulties, fatigue, and, in rare cases, seizures or subdural fluid collections (3, 4). Such observations challenge the earlier assumption that PDPH is uniformly short-lived and clinically uncomplicated.

Long-term follow-up data further support this more differentiated perspective. In a 5-year follow-up study of women with unintentional dural puncture during initiation of epidural labor analgesia, approximately 20% reported persistent chronic headache irrespective of EBP treatment, accompanied by similar rates of chronic back pain and substantial functional impairment (5). Previous retrospective studies in obstetric patients have reported persistent headache in 28–34% at 18 months after unintended dural puncture (6). A meta-analysis including 6,541 obstetric patients demonstrated a nearly fourfold increased relative risk of chronic headache persisting ≥12 months after unintentional dural puncture and/or PDPH (RR 3.95), along with elevated risks of back pain, neck pain, and depressive symptoms (7). However, absolute long-term event rates varied considerably across studies, reflecting heterogeneity in design, follow-up duration, and outcome definitions. Within this broader context, delayed and refractory courses following obstetric neuraxial anesthesia have also been described. In some patients, headache develops weeks to years after delivery and may initially resemble a primary headache disorder. Neuroimaging in these cases frequently demonstrates persistent CSF leakage or imaging features consistent with intracranial hypotension (8). Nevertheless, systematically collected longitudinal data in this subgroup remain limited.

Despite emerging evidence of persistent morbidity, data on surgically treated PDPH remain particularly scarce. The available literature consists largely of small cohort studies, case reports and case series, resulting in a low level of evidence and limiting robust conclusions regarding management strategies and long-term outcomes (4). In addition, patients with persistent symptoms after PDPH increasingly seek information and peer support through patient communities and online platforms. Although these platforms do not provide epidemiological data, they suggest the presence of a patient population whose postoperative course and long-term symptom burden have not been systematically characterized in the scientific literature.

Against this background, the present study aimed to provide structured descriptive data on symptom burden, functional status, and postoperative trajectories among respondents recruited through online patient communities who self-reported prior surgical treatment for persistent symptoms attributed to PDPH or CSF leak following dural puncture.

## Methods

### Study design and recruitment

This study was conducted as an online cross-sectional survey between 1 February and 1 April 2025. The study protocol was reviewed and approved by the Ethics Committee of the Medical University of Vienna (approval number 1111/2025). Electronic informed consent was obtained from all participants prior to survey initiation. Data collection was performed using the data protection–compliant platform SoSci Survey.^1^ To prevent automated entries, reCAPTCHA verification was implemented, and duplicate protection was activated to block multiple submissions from the same device or IP address. The study was reported in accordance with the STROBE (Strengthening the Reporting of Observational Studies in Epidemiology) guidelines for cross-sectional studies and the CHERRIES checklist for web-based surveys (9, 10).

The present analysis focuses exclusively on respondents who self-reported prior surgical treatment for PDPH or CSF leak following dural puncture. Participants were recruited through established international digital self-help groups and patient-led organizations dedicated to CSF leaks and intracranial hypotension. Recruitment channels included the Spinal CSF Leak Foundation (USA), the CSF Leak Association (UK), CSF Leak Canada, the Spanish-speaking Asociación AHIFUGA, as well as several international Facebook support groups for individuals with PDPH, iatrogenic CSF leaks, and intracranial hypotension.

These platforms primarily function as peer-support networks that facilitate exchange of experiences and provide informational and emotional support throughout often prolonged diagnostic and therapeutic pathways. They were used solely as recruitment channels to access a geographically dispersed and otherwise difficult-to-reach patient population. No assumptions regarding disease prevalence, incidence, or representativeness were derived from these communities.

### Questionnaire development and patient involvement

The questionnaire was developed in collaboration with administrators of relevant self-help groups, some of whom have medical backgrounds or personal experience with PDPH. This participatory approach aimed to ensure that the survey content was clinically relevant while remaining comprehensible and grounded in patient experience. Such involvement aligns with current recommendations for Patient and Public Involvement (PPI) in health research and enhances the relevance and face validity of collected data. To improve data quality, automated completeness checks were implemented. Participants were encouraged to consult available medical reports or physician letters when answering questions regarding surgical procedures and diagnostic findings; however, no formal document verification was performed.

### Study population

The survey formed part of a broader investigation of iatrogenic CSF leaks. For this study, the analytical cohort comprised respondents who answered ‘yes’ to the screening question, ‘Have you undergone surgery for PDPH or a CSF leak following a dural puncture?’. Eligible participants were required to be at least 18 years of age and to self-report prior surgical treatment for persistent symptoms attributed to PDPH or CSF leak following dural puncture. In addition, participants were required to have sufficient English language proficiency to complete the questionnaire independently and to provide electronically documented informed consent prior to participation in the study. Diagnoses, leak location, surgical indications, operative procedures, and technical success were based on respondent report and were not independently adjudicated. Filter logic was applied to exclude respondents who did not meet eligibility criteria. Incomplete responses—particularly those limited to demographic information were excluded from final analyses.

### Questionnaire structure

The full version of the questionnaire is provided as Supplementary file 1. The questionnaire comprised the following domains:

- Sociodemographic characteristics: Age, sex, anthropometric measures, ethnicity, marital status, education level, employment status, and income were recorded.
- Clinical characteristics: Information was collected on headache history prior to PDPH, including pre-existing primary headache disorders, baseline headache frequency, and relevant comorbidities (e.g., Ehlers–Danlos syndrome, postural orthostatic tachycardia syndrome, ME/CFS, Chiari malformation). Participants also reported the cause of dural puncture or CSF leak, including diagnostic or therapeutic lumbar puncture and epidural anesthesia.
- Surgical treatment and course: Participants were asked to provide detailed information regarding their surgical management and postoperative course. This included the total number of CSF leak–related surgeries performed and the spinal region involved in surgery, recorded using predefined categorical level labels: cervical, thoracic, lumbar, or other. In addition, participants reported the time interval between diagnosis and their most recent surgery, the elapsed time since the most recent surgical intervention, and the duration of postoperative sick leave. They were further asked to characterize their perceived postoperative symptom trajectory (e.g., complete resolution, partial improvement, stable symptoms, recurrence, or worsening) and to describe their current postoperative headache phenotype, including persistent orthostatic headache, features suggestive of rebound intracranial hypertension, diffuse non-specific headache, or complete remission.
- Functional and occupational consequences: Items assessed return to work, reduced working hours, unemployment, health-related retirement, and ongoing medical leave, as well as general limitations in daily functioning.
- Psychosocial impact: Current psychological symptoms (e.g., depressed mood, anxiety, sleep disturbance, fatigue, cognitive difficulties), use of psychological therapy, perceived emotional burden, effects on personal relationships, and type of healthcare cost coverage were documented.

The questionnaire also collected information on diagnostic workup, postoperative imaging, and treatment modalities; these domains were outside the scope of the present analysis and are addressed in a separate manuscript currently under review.

### Headache-related disability

Headache-related disability was assessed using the Headache Impact Test-6 (HIT-6), a validated instrument measuring headache-associated impairment in daily life. The HIT-6 consists of six items evaluating pain intensity, social functioning, cognitive performance, and emotional burden. Total scores range from 36 to 78, with higher scores indicating greater disability. Based on established thresholds, four categories were distinguished: ≤49 (little or no impact), 50–55 (moderate impact), 56–59 (substantial impact), and ≥60 (severe impact).

### Sample size

No formal *a priori* sample size calculation was performed due to the absence of reliable data for effect size estimation in surgically treated PDPH populations. An exploratory design was therefore chosen, and all available eligible responses were included in the analysis.

### Statistical analysis

Analyses were conducted descriptively due to the exploratory study design and small subgroup sizes. HIT-6 scores were analysed as both continuous and categorical variables. Established cut-offs (≤49, 50–55, 56–59, ≥60) were applied for clinical classification and were additionally reflected in color-coded graphical presentations. For each subgroup, HIT-6 scores are reported as mean ± standard deviation (SD) to describe central tendency and dispersion. Categorical variables are presented as n (%), and non-normally distributed continuous variables as median (IQR). All statistical analyses were conducted using SPSS version 28.0 (IBM Corp., Armonk, NY). For the creation of the presented figures, GraphPad Prism 10 for Windows, version 10.2.2 (341), was employed. All items were fully completed by all participants, with no missing values in the dataset.

## Results

A total of 582 individuals accessed the online questionnaire, of whom 302 initiated the survey. Of these, 48 were excluded at initial screening: 27 reported no prior surgical treatment and 21 reported skull-base CSF leaks outside the scope of the survey, leaving 254 respondents who met the eligibility criteria. A further 26 discontinued the survey before completion and were excluded due to incomplete data. The final cohort comprised 228 respondents, of whom 136 reported prior surgical treatment for PDPH or iatrogenic CSF leak following dural puncture and 92 reported spontaneous intracranial hypotension; the present analysis is restricted to the former group.

In Table 1, sociodemographic characteristics stratified by HIT-6 group are presented. A total of 136 respondents who self-reported prior surgical treatment for PDPH or CSF leak following dural puncture were included; 35 (25.7%) had HIT-6 scores ≤49 and 101 (74.3%) had scores ≥50. Sex distribution was comparable between groups, with females representing 77.1% of the ≤49 group and 72.3% of the ≥50 group. Median age was similar between groups (39 [IQR 35–44] vs. 38 [33–42] years). The median time from diagnosis to the most recent CSF leak surgery was 27 (13–63) months in the ≤49 group and 25 (13–38) months in the ≥50 group. Median elapsed time since surgery was 20 (14–26) months and 18 (13–23) months, respectively. Median sick leave duration following the most recent surgery was 6 (4–10) months in the ≤49 group and 8 (4–11) months in the ≥50 group. Further sociodemographic and clinical details are provided in Table 1.

**Table 1:** Sociodemographic characteristics by HIT-6 group.

Variable	Group ≤49 (*n* = 35)	Group ≥50 (*n* = 101)
Sex, *n* (%)		
Male	8 (22.9)	28 (27.7)
Female	27 (77.1)	73 (72.3)
Age (years), Median (IQR)	39 (35–44)	38 (33–42)
Anthropometric measures, Median (IQR)		
Weight (kg)	70 (57–80)	64 (57–79)
Height (cm)	172 (165–177)	168 (163–174)
Ethnicity, *n* (%)		
White/Caucasian	22 (62.9)	76 (75.2)
Black/African American	1 (2.9)	4 (4.0)
Latin American	4 (11.4)	8 (7.9)
Asian	5 (14.3)	10 (9.9)
Arab/Middle Eastern	3 (8.6)	3 (3.0)
Marital status, *n* (%)		
Married	7 (20.0)	21 (20.8)
Divorced	6 (17.1)	25 (24.8)
Single	14 (40.0)	34 (33.7)
Widowed	2 (5.7)	0 (0)
In a relationship	6 (17.1)	16 (15.8)
Prefer not to answer	0 (0)	5 (5.0)
Level of education, *n* (%)		
Less than high school	4 (11.4)	16 (15.8)
High school or some college	6 (17.1)	30 (29.7)
Bachelor’s degree	10 (28.6)	34 (33.7)
Postgraduate degree (e.g., Master’s, PhD)	15 (42.9)	21 (20.8)
Net income, *n* (%)		
<€500	1 (2.9)	5 (5.0)
€500–1,000	2 (5.7)	5 (5.0)
€1,000–1,500	2 (5.7)	15 (14.9)
€1,500–2,000	8 (22.9)	9 (8.9)
€2,000–3,000	5 (14.3)	21 (20.8)
€3,000–4,000	7 (20.0)	10 (9.9)
€4,000–5,000	0 (0)	6 (5.9)
≥€5,000	2 (5.7)	6 (5.9)
Prefer not to answer	8 (22.9)	24 (23.8)
Professional status, *n* (%)		
Training/apprenticeship	0 (0)	1 (1.0)
Employee	28 (80.0)	59 (58.4)
Unemployed/seeking employment	3 (8.6)	18 (17.8)
Retired (age-related)	0 (0)	1 (1.0)
Retired due to chronic PDPH	0 (0)	8 (7.9)
Self-employed	0 (0)	4 (4.0)
In school/university	1 (2.9)	5 (5.0)
Prefer not to answer	3 (8.6)	5 (5.0)
Surgical treatment characteristics, months, median (IQR)		
Time from diagnosis to most recent CSF leak surgery	27 (13–63)	25 (13–38)
Elapsed time since CSF leak surgery	20 (14–26)	18 (13–23)
Sick leave duration since recent CSF leak surgery	6 (4–10)	8 (4–11)

Categorical variables presented as *n* (). Continuous variables presented as Median (IQR = P25–P75). HIT-6 dichotomized using validated cut-off: ≤49 = no substantial headache impact; ≥50 = substantial headache impact.

**Figure 1:**
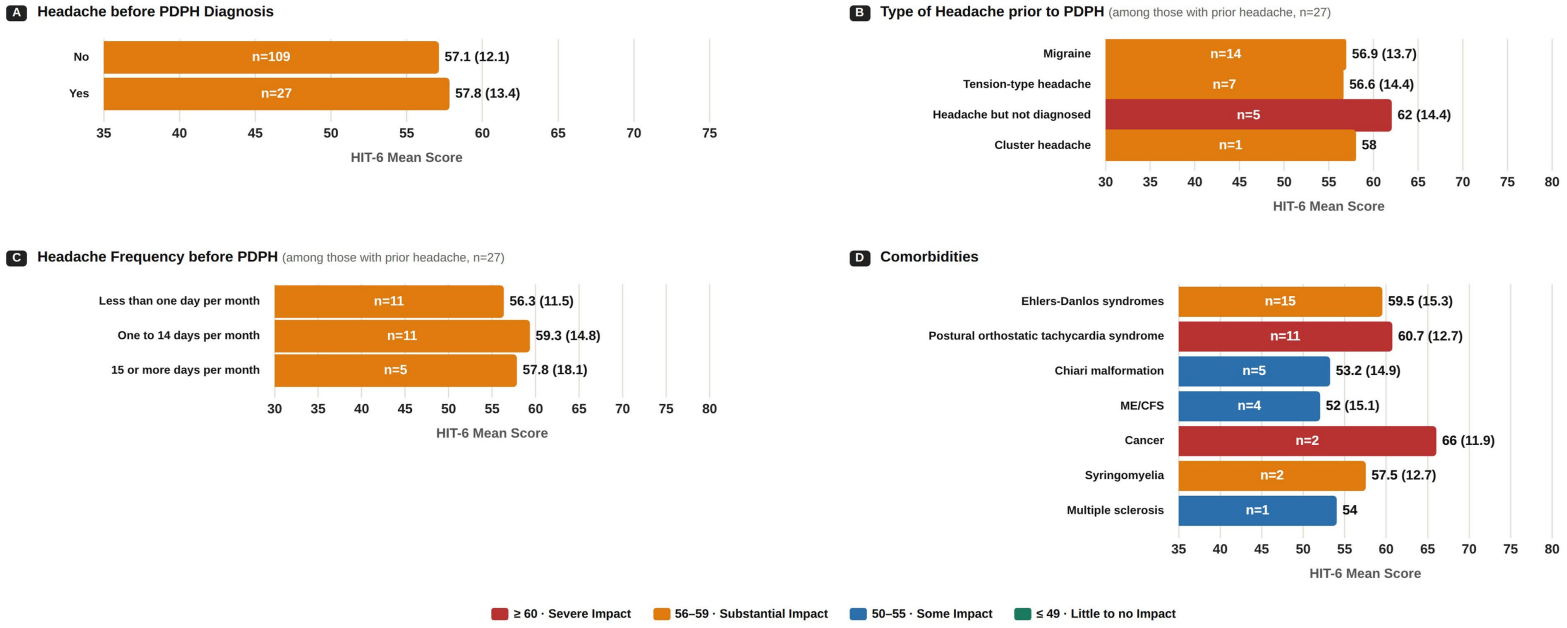
Preoperative headache characteristics in relation to current HIT-6 scores. Bars show mean HIT-6 (Headache Impact Test-6) scores ± SD. HIT-6 categories: ≤49 little/no impact; 50–55 moderate; 56–59 substantial; ≥60 severe. **(A)** Headache before PDPH diagnosis; **(B)** type of headache prior to PDPH; **(C)** headache frequency before PDPH; **(D)** comorbidities. PDPH = post-dural puncture headache; ME/CFS = myalgic encephalomyelitis/chronic fatigue syndrome.

As shown in Figure 1, prior to PDPH diagnosis, 27 participants (20.0%) reported pre-existing primary headaches. Among these, migraine was reported by 14 (51.9%), tension-type headache by 7 (25.9%), headache without clinician diagnosis by 5 (18.5%), and cluster headache by 1 (3.7%). Mean HIT-6 scores in these subgroups were 56.9 (SD 13.7) for migraine, 56.6 (14.4) for tension-type headache, 62.0 (14.4) for headache without clinician diagnosis, and 58.0 (13.8) for cluster headache. Regarding comorbidities, Ehlers-Danlos syndromes were reported by 15 participants (11.0%) with a mean HIT-6 of 59.5 (15.3), postural orthostatic tachycardia syndrome by 11 (8.1%) with a mean of 60.7 (12.7), Chiari malformation by 5 (3.7%) with a mean of 53.2 (14.9), and ME/CFS by 4 (2.9%) with a mean of 52.0 (15.1).

**Figure 2:**
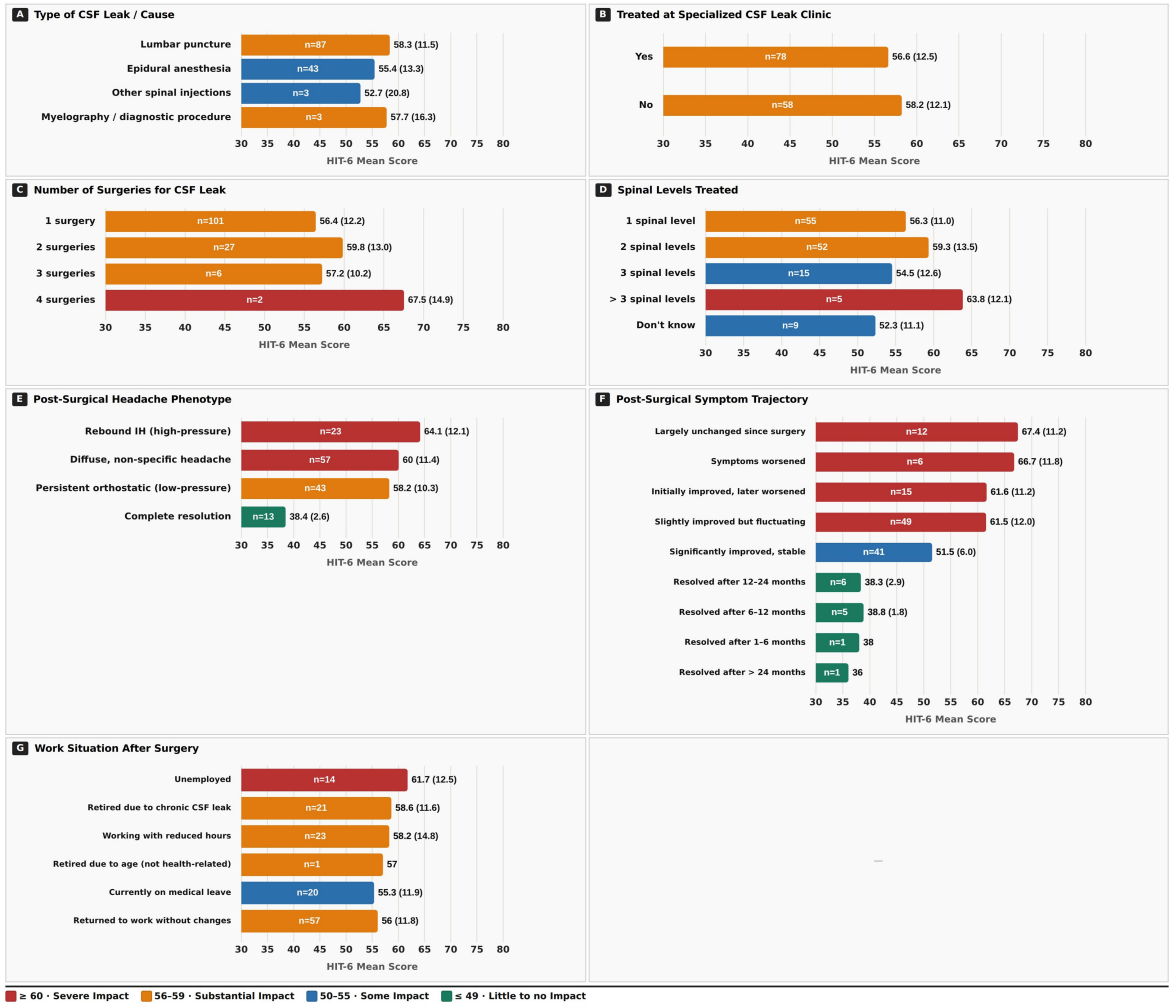
CSF leak etiology, surgical factors, and postoperative outcomes in relation to HIT-6 scores. Bars show mean HIT-6 ± SD. HIT-6 categories: ≤49 little/no impact; 50–55 moderate; 56–59 substantial; ≥60 severe. CSF = cerebrospinal fluid; IH = intracranial hypertension. **(A)** Type of CSF leak/cause; **(B)** treated at specialized CSF leak clinic; **(C)** number of surgeries; **(D)** spinal levels treated; **(E)** post-surgical headache phenotype; **(F)** post-surgical symptom trajectory; **(G)** work situation after surgery.

Figure 2 provides an overview of reported CSF leak causes, surgical characteristics, postoperative headache phenotypes, symptom trajectories, and work status. Diagnostic or therapeutic lumbar puncture was the most common reported cause of CSF leak (65.1%), followed by epidural anesthesia (30.6%). Most respondents had undergone one surgical intervention (73.1%), and the lumbar spine was the most commonly reported surgical level. Slight and fluctuating improvement was the most common postoperative trajectory, whereas complete remission was reported by 9.6% of respondents. HIT-6 scores differed across headache phenotypes and work-status categories, with higher mean scores among respondents reporting rebound intracranial hypertension, diffuse non-specific headache, unemployment, or ongoing medical leave.

**Figure 3:**
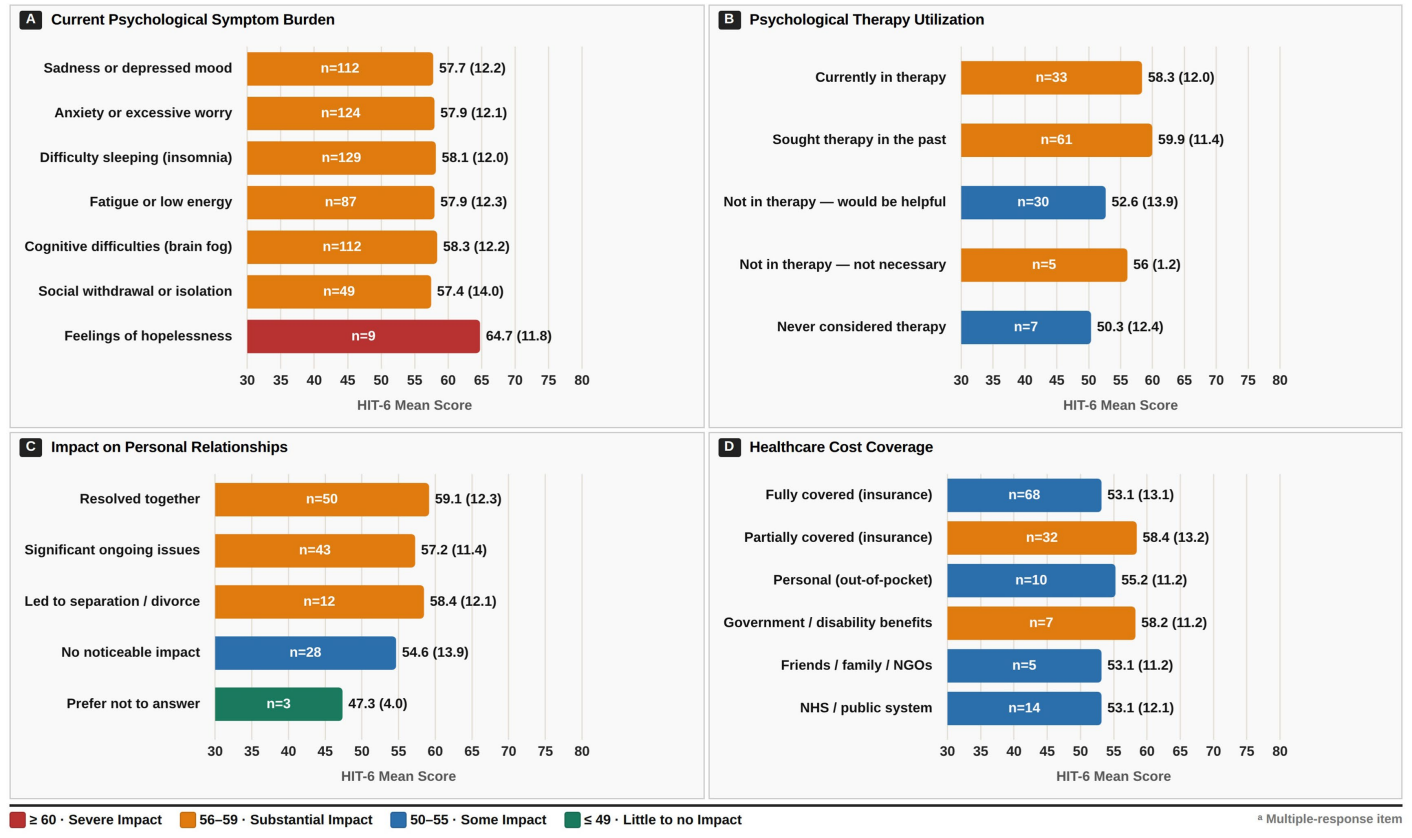
Psychological symptom burden, therapy utilization, social impact, and cost coverage in relation to HIT-6 scores. Bars show mean HIT-6 ± SD. HIT-6 categories: ≤49 little/no impact; 50–55 moderate; 56–59 substantial; ≥60 severe. NHS = National Health Service; NGO = non-governmental organization. **(A)** Current psychological symptom burden; **(B)** psychological therapy utilization; **(C)** impact on personal relationships; **(D)** healthcare cost coverage.

As shown in Figure 3, psychological symptoms were frequently reported. Difficulty sleeping (94.9%), anxiety (91.2%), sadness or depressed mood (82.4%), and cognitive difficulties (82.4%) were most common. Mean HIT-6 scores across these symptom groups were similar (approximately 58). Feelings of hopelessness were less frequent (6.6%) and showed a higher mean HIT-6 (64.7 [SD 11.8]). Regarding psychological therapy utilization, 24.3% were currently in therapy and 44.9% had sought therapy in the past. With respect to relationship impact, 36.8% reported resolved difficulties and 31.6% ongoing issues.

## Discussion

In this exploratory cross-sectional analysis of respondents who self-reported prior surgical treatment for PDPH or CSF leak following dural puncture, substantial headache-related disability emerged as a key finding, with approximately three quarters of respondents recording HIT-6 scores ≥50, consistent with at least moderate headache impact. Because diagnoses, surgical details, technical success, postoperative phenotype, and symptom trajectories were self-reported and not independently verified, the findings are best interpreted as patient-reported postoperative burden rather than adjudicated surgical outcomes. Nevertheless, the data provide robust evidence of substantial headache-related disability within this surveyed cohort. Approximately one fifth of participants reported pre-existing primary headache disorders prior to PDPH onset, most frequently migraine and tension-type headache. Mean current HIT-6 scores in these subgroups were broadly comparable to those of the overall cohort. Although the study design does not allow determination of the extent to which prior headache vulnerability contributed to current symptom burden, these findings indicate that substantial current headache-related disability was not restricted to respondents with self-reported pre-existing primary headache disorders. Prior literature has proposed an association between migraine and altered CSF dynamics, with the hypothesis that patients with migraine may exhibit heightened sensitivity to changes in intracranial pressure (11). However, findings from the EPiMAP obstetric cohort suggest that the role of migraine history may be more nuanced: Gupta et al. (12) reported that migraine history was associated with a higher likelihood of receiving a second epidural blood patch, but not necessarily with greater headache intensity after the procedure. Whether such a predisposition modulates recovery after surgical CSF leak repair remains an open question that prospective studies with adequate longitudinal follow-up should address.

Beyond the question of pre-existing headache vulnerability, the postoperative symptom trajectories themselves reveal a clinically important and poorly understood degree of heterogeneity. Within the surveyed cohort, complete remission was reported by approximately 10% of participants; most respondents in this subgroup reported full recovery between 6 months and 2 years after the most recent surgical intervention. This temporal pattern raises the possibility that a subset of patients currently categorized as persistently symptomatic may represent a transitional state rather than a fixed outcome, and that extended follow-up intervals may be necessary to capture the full range of postoperative trajectories. Approximately one third of participants reported significant but persistent improvement, with mean HIT-6 scores of approximately 51.5, indicating ongoing moderate impairment that, while clinically meaningful, falls below the threshold for severe disability. Whether this subgroup represents a stable endpoint or a phase of continued, albeit slow, recovery cannot be determined from the present data.

The largest single subgroup, comprising approximately 39% of the cohort, experienced only slight and fluctuating improvement, with a mean HIT-6 of approximately 61.5 reflecting continued substantial burden. Notably, more than half of the overall cohort recorded HIT-6 scores ≥60, corresponding to severe headache-related disability. Taken together, more than 90% of respondents in this self-selected cohort reported clinically relevant symptoms at the time of assessment, underscoring the need to assess patient-reported symptom burden beyond the surgical intervention itself and to incorporate such outcomes into postoperative follow-up. This pattern raises a central and currently unresolved question: which factors are associated with full or near-complete remission versus persistent symptom burden following surgical intervention? No systematic studies have examined predictors of postoperative outcome in surgically treated PDPH populations. The available evidence is confined to case-level reports, which nonetheless illustrate the clinical range of possible trajectories in an instructive manner. In one reported case, surgical dural repair performed after more than 2 years of refractory PDPH resulted in immediate and sustained resolution of symptoms, a course consistent with a persistent structural dural defect as the dominant pathophysiological driver (13). In contrast, a case describing microsurgical repair of a low-flow arachnoid bleb arising from a lumbar puncture site demonstrated only slow and partial symptom improvement over several months, despite intraoperative confirmation of active CSF leakage (14). These divergent outcomes underscore that structural closure of a dural defect does not uniformly translate into clinical remission, and that the relationship between anatomical repair and symptom resolution is likely mediated by additional, poorly characterized factors.

The mechanisms underlying persistent symptoms after surgical treatment remain uncertain and were not directly assessed in the present survey. Persistent or recurrent CSF leakage, altered CSF pressure regulation, and headache chronification mechanisms such as central sensitization remain plausible, hypothesis-generating explanations (1, 15, 16). This interpretation is consistent with previous clinical observations that symptoms persisting after a dural puncture cannot always be explained by a simple model of ongoing CSF loss. In their cohort study, Fung et al. found no consistent evidence of CSF hypotension or significant leakage in a large proportion of patients with chronic PDPH (17). Similarly, Schievink et al. reported that digital subtraction myelography identified a treatable structural lesion in only 9 of 27 patients with refractory chronic PDPH (18). These findings support the concept that persistent symptom burden after dural puncture may reflect heterogeneous mechanisms, including persistent leakage, altered CSF dynamics, and central sensitisation. The primary contribution of the present study lies in providing a structured characterization of patient-reported headache disability, functional status, symptom trajectories, and psychosocial burden in a population that is systematically underrepresented in conventional clinical series.

Comparison with outcomes in surgically treated spontaneous intracranial hypotension (SIH) may offer some orientation, but it has clear methodological limitations. SIH is a different clinical condition with distinct leak types, often located in the thoracic or cervicothoracic spine, and a pathophysiology that differs from iatrogenic dural puncture. Therefore, findings from surgical SIH cohorts cannot be directly transferred to patients with PDPH and should be interpreted with caution. Nevertheless, data from Volz et al. (19) are informative in this context: the authors reported a reduction in median HIT-6 score from 65 preoperatively to 49 postoperatively in a cohort of surgically treated SIH patients, reflecting a clinically meaningful but incomplete improvement. Critically, only 39% of the original cohort remained in follow-up at 12 months, leaving the postoperative trajectories of the majority of patients uncharacterized. This substantial loss to follow-up limits interpretation, as attrition could operate in either direction. Patients with unfavorable outcomes may be lost to follow-up, potentially leading to overestimation of treatment effects. Conversely, patients with complete resolution may disengage from follow-up, potentially leading to underestimation of favorable outcomes.

The high prevalence of psychological symptoms in the present cohort, including sleep disturbance, anxiety, depressed mood, and cognitive difficulties, each affecting a clear majority of participants, is notable. This pattern resembles constellations described in other chronic and difficult to treat pain conditions, in which psychological and functional impairments frequently co-occur (20). Whether the observed symptoms represent consequences of persistent headache burden, reflect shared underlying factors, or arise from bidirectional reinforcement cannot be determined from the present cross-sectional data. Bidirectional associations between headache severity, sleep disturbance, and affective symptoms have been described in the broader chronic headache literature (20).

The documented relational consequences further highlight the broader impact of the condition. Approximately one third of participants reported ongoing significant difficulties in personal relationships, and 8.8% indicated that their illness had contributed to separation or divorce. Similar observations were reported by Kraus et al. in a cohort of 30 patients with chronic PDPH, in which marked impairments in social and relational functioning were also described. The present data suggest that such psychosocial burdens may persist even in surgically treated patients (16). The high utilization of psychological therapy, with nearly 70% of participants reporting current or past engagement in psychological treatment, indicates that a substantial proportion perceived a need for professional support. Whether this primarily reflects symptom severity or structural gaps in care cannot be determined from the available data.

Several limitations must be carefully considered when interpreting these findings. The cross-sectional design does not allow causal inferences, and the absence of preoperative baseline data prevents direct assessment of changes in headache-related disability following surgery. Prospective longitudinal studies with repeated assessments are necessary to better characterize postoperative trajectories and potential treatment effects. Recruitment through international online patient self-help groups represents a major limitation and may have introduced substantial selection bias. Individuals with persistent, complex, or unsatisfactory postoperative courses are more likely to remain active in such communities and therefore to participate in the survey. Conversely, patients who achieved full or near-complete remission may disengage from support groups and are likely underrepresented. As a result, the reported prevalence and severity of long-term symptom burden may not reflect the broader population of surgically treated PDPH patients. In addition, only surgically treated individuals were included, and the findings cannot be generalized to patients managed conservatively or with epidural blood patch alone. All data were based on self-report and were not independently verified against medical records or operative documentation. Therefore, diagnoses, leak location, surgical indications, operative procedures, and technical success could not be independently adjudicated. Misclassification, recall bias, or misunderstanding of clinical details cannot be excluded. Furthermore, the use of an English-language questionnaire may have limited participation of non-English-speaking individuals. Notwithstanding these limitations, this study provides structured descriptive data on patient-reported burden in a self-selected cohort reporting prior surgical treatment for PDPH or CSF leak following dural puncture, capturing a population that is systematically underrepresented in conventional clinical series.

## Conclusion

The present study provides structured descriptive data from an international online support-group cohort of respondents reporting prior surgical treatment for PDPH or CSF leak following dural puncture. Within this cohort, persistent headache burden and psychosocial impairment were frequently reported and affected a large proportion of respondents, while complete symptom remission was reported by a minority. Postoperative symptom trajectories were highly variable, highlighting the need for prospective studies with improved, structured, and continuous long-term follow-up to enable more accurate characterization of recovery patterns and the development of evidence-based follow-up frameworks for this underserved population.

## Data Availability

The raw data supporting the conclusions of this article will be made available by the authors, without undue reservation.

## Footnotes

1
https://www.soscisurvey.de
