# Supplementary material for: Persistent headache burden after surgical treatment of post-dural puncture headache: a cross-sectional study
Source: Front Neurol. 2026 May 25;17:1846001. doi: 10.3389/fneur.2026.1846001 (PMC13243079; doi:10.3389/fneur.2026.1846001)
Supplement: Supplementary file 1 [file Table_1.docx]

Version 1, 23.01.2025

**Questionnaire: Experience, outcomes and long-term effects after surgery in individuals with cerebrospinal fluid leaks**

**Personal Information**

**1. Have you been treated surgically for a cerebrospinal fluid leak (CSF leak)?**
☐ Yes
☐ No

**If yes, what type of CSF leak were you diagnosed with?**
☐ Spontaneous intracranial hypotension (SIH)
☐ Iatrogenic CSF leak (caused by medical intervention)

**If iatrogenic CSF leak, what was the cause?**
☐ Lumbar puncture for diagnostic or therapeutic purposes
☐ Epidural anesthesia
☐ Spinal surgery
☐ Other injections in the spinal region
☐ Other: __________

**2. What is your biological sex?**
☐ Male
☐ Female

**3. How old are you?**
I am ____ years old.

**4. What is your weight in kilograms?**
__________ kg

**5. What is your height in centimeters?**
__________ cm

**6. What is your ethnicity?**
☐ White / Caucasian
☐ Black / African American
☐ Latin American
☐ Asian
☐ Arab / Middle Eastern
☐ Other: __________

**7. What is your marital status?**
☐ Married
☐ Divorced
☐ Single
☐ Widowed
☐ In a relationship
☐ Prefer not to answer

**8. What is the highest level of education you have completed?**
☐ Less than high school
☐ High school or some college
☐ Bachelor’s degree
☐ Postgraduate degree (e.g., Master’s, PhD)

**9. What do you do professionally?**
☐ Training / apprenticeship
☐ Employee
☐ Unemployed / seeking employment
☐ Retired
☐ Self-employed
☐ University student
☐ In school
☐ Prefer not to answer

**10. What is your monthly net income?**
☐ Less than €500
☐ €500 to €1,000
☐ €1,000 to €1,500
☐ €1,500 to €2,000
☐ €2,000 to €3,000
☐ €3,000 to €4,000
☐ €4,000 to €5,000
☐ €5,000 or more
☐ Prefer not to answer

**11. In which country are you currently living?**

**Medical History and Workability**

**12. Have you been diagnosed with any other significant medical condition?**
☐ Cancer
☐ Diabetes
☐ Kidney disease
☐ Stroke
☐ Parkinson’s disease
☐ Postural orthostatic tachycardia syndrome (POTS)
☐ Syringomyelia
☐ Chiari malformation
☐ Ehlers-Danlos syndromes (EDS)
☐ Multiple sclerosis (MS)
☐ Liver disease
☐ Chronic obstructive pulmonary disease (COPD)
☐ Heart disease
☐ ME/CFS
☐ Other: __________
☐ Other: __________
☐ Other: __________

**13. For how long have you been on sick leave after the surgery for your CSF leak?**
☐ _____ weeks
☐ None

**14. How many hours per week, on average, have you been able to work after the surgery for your CSF leak?**
☐ Full-time (30–40 hours per week)
☐ Part-time (10–20 hours per week)
☐ I had to change my job due to chronic CSF leak
☐ I am no longer able to work due to a chronic CSF leak
☐ Other: __________

**15. Have you experienced any type of headache prior to the onset of your CSF leakage?**
☐ No
☐ Yes

**16. Have you been diagnosed by a physician with any of the following headache disorders?**
☐ Tension-type headache
☐ Cluster headache
☐ Migraine
☐ Other diagnosis (please specify): __________

**17. If yes, how frequently did you experience headaches in the three months leading up to the onset of your CSF leakage?**
☐ Less than one day per month
☐ One to 14 days per month
☐ 15 or more days per month

**18. Is the headache still orthostatic after the CSF leak surgery?**
(Worse when standing, better when lying down)
☐ Yes
☐ No

**19. Have you developed new headaches after the surgery that are different from the ones before?**
☐ Yes
☐ No

**20. Has a physician diagnosed you with intracranial rebound hypertension?**
☐ Yes
☐ No
☐ Not applicable

**If yes, how was the intracranial rebound hypertension diagnosed?**
(Check all that apply)
☐ Magnetic resonance imaging (MRI, a scan that uses magnets and radio waves to create detailed images of your brain)
☐ Computed tomography (CT scan, an advanced X-ray that provides detailed pictures of your head)
☐ Lumbar puncture for pressure measurement (a procedure where a small amount of cerebrospinal fluid is taken from your spine to check the pressure)
☐ Other diagnostic procedure (please specify): _______________

**21. How would you describe your headaches?**
☐ Dull
☐ Sharp
☐ Pressing
☐ Pulsating
☐ Burning
☐ Other: _______________

**22. Where are the headaches located?**
☐ Temples
☐ Forehead
☐ Back of the head
☐ Neck region
☐ Whole head
☐ Other: _______________

**23. Do the headaches change location or remain constant?**
☐ The location of the pain varies.
☐ The intensity of the pain varies.
☐ The pain remains constant.

**24. How severe are the headaches on a scale from 0 to 10?**
(0 = no pain, 10 = the worst pain imaginable)
Intensity: ________

**25. How has the frequency or intensity of the headaches changed since the surgery?**
☐ Improved
☐ Worsened
☐ Stayed the same

**26. How long do the headaches usually last?**
☐ A few minutes
☐ Hours
☐ All day
☐ Other: _______________

**27. Are there specific triggers or factors that worsen the headaches?**
Triggers: _______________

**28. Have you received any treatments or medications for the headaches?**
☐ Yes, please specify: _______________
☐ No

**29. How effective have the treatments been so far?**
☐ No improvement
☐ Slight improvement
☐ Significant improvement
☐ Complete resolution of the pain

**30. Do you experience additional symptoms with the headaches?**
(Check all that apply)
☐ Nausea
☐ Vomiting
☐ Tinnitus (ringing in the ears)
☐ Dizziness
☐ Sensitivity to light
☐ Sensitivity to sound
☐ Neck stiffness
☐ Pain at the surgical site (e.g., back or spine)
☐ Lower back pain
☐ Shoulder or upper back pain
☐ Fatigue or exhaustion
☐ Brain fog (difficulty thinking clearly)
☐ Concentration difficulties
☐ Memory issues
☐ Balance problems or feeling unsteady
☐ Visual disturbances (e.g., blurred vision, double vision)
☐ Tingling or numbness in extremities
☐ Other: _______________

31. Compared to the time of onset of your CSF leakage, what describes your current condition best?
☐ Complete symptom resolution
☐ Significant sustained improvement
☐ Mild improvement with fluctuations
☐ No change in symptom severity
☐ Initial improvement with subsequent relapse
☐ Symptom worsening since surgery

**32. When was your surgery for the CSF leak performed?**
_____________ week(s) ago

**33. How many surgeries have you undergone for your CSF leak?**
_________ surgery

**34. Have you undergone surgery at a centre specialising in treating leaking CSF?**
☐ Yes
☐ No

**35. Where was the CSF leak located?**
☐ Cervical spine
☐ Thoracic spine
☐ Lumbar spine
☐ Other: _______________

36. What type of spinal CSF leak were you diagnosed with?
☐ Ventral dural CSF leak
☐ Dorsal dural CSF leak
☐ Lateral CSF leak
☐ CSF-venous fistula
☐ I don’t know
☐ Other: __________

**37. How long was the time from diagnosis of your CSF leak (SIH) to your most recent surgery?**
_____ months

**38. How much time has passed since your most recent CSF leak surgery?**
_____ months

**39. Have structural abnormalities related to your CSF leak been identified (e.g., on imaging or during surgery)?**
☐ No
☐ Yes
☐ I don’t know

**If yes, which of the following were identified? (Check all that apply)**
☐ Pseudomeningocele
☐ Epidural neo-membranes
☐ Combined pseudomeningocele and neo-membranes
☐ CSF-venous fistula
☐ Other structural abnormality: __________

**40. Did you experience any complications during surgery for your CSF leak?**
(Check all that apply)
☐ Excessive blood loss
☐ Dural tear or additional damage to the dura
☐ Nerve damage (e.g., sensory or motor impairment)
☐ Difficulty in sealing the leak (e.g., incomplete closure)
☐ Anaesthesia-related complications (e.g., breathing difficulties, allergic reactions)
☐ CSF-venous fistula identified and treated during surgery
☐ Other intraoperative complications (please specify): __________

**41. How long did you stay in the hospital after the surgery?**
_____ days

**42. Which diagnostic tests were performed after surgery?**
☐ MRI brain
☐ MRI spine
☐ CT myelography
☐ Dynamic subtraction myelography (DSM)
☐ Ultrasound optic nerve sheath
☐ Other: __________

**43. Did your physicians use the Bern SIH Score?**
☐ Yes
☐ No
☐ I don’t know

**44. Total Bern SIH Score:**
☐ 0–1
☐ 2–3
☐ ≥4
☐ Unknown

**45. Which factors worsen your symptoms?**
(Check all that apply)
☐ Weather changes
☐ Physical activity
☐ Stress or emotional state
☐ Sleep quality
☐ Infections
☐ Sodium intake
☐ Hormonal changes (e.g., menstrual cycle)
☐ Other: __________

**46. What is your current employment status after CSF leak surgery?**
☐ Returned to work without changes (same hours, same position)
☐ Currently on medical leave since surgery
☐ Working with reduced hours due to persistent symptoms
☐ Medically retired due to chronic CSF leak
☐ Unemployed (not due to health reasons)
☐ Retired due to age (not health-related)
☐ Other: __________

**47. Do you feel afraid to engage in physical activity after surgery?**
☐ Yes
☐ No

**49. Did the illness cause any difficulties in your relationships?**
☐ Yes, it caused problems, but we worked through them.
☐ Yes, it caused significant issues, and we are still struggling.
☐ Yes, it caused significant issues, and we ultimately decided to separate or divorce.
☐ No, it did not have any noticeable impact.
☐ Prefer not to answer

**50. Do you believe that the CSF leak and its consequences may have caused emotional distress?**
(Check one)
☐ No
☐ Yes, I feel emotionally distressed and experience significant psychological strain.
☐ Yes, I feel emotionally affected, but I’m unsure if it qualifies as significant distress.
☐ I prefer not to answer

**51. Which of the following psychological symptoms are you currently experiencing?**(Check all that apply)
☐ Persistent sadness or hopelessness
☐ Frequent anxiety or fear
☐ Difficulty coping with everyday responsibilities or stressors
☐ Recurrent thoughts of self-harm or suicide
☐ Prefer not to answer

**52. Have you considered or sought psychological therapy to help cope with your condition?**
(Check one)
☐ Yes, I am currently in psychological therapy.
☐ Yes, I have sought therapy in the past.
☐ No, but I feel this type of support would be helpful.
☐ No, I do not feel this type of support is necessary.

☐ No, I have not considered psychological support

**53. Have you been able to increase your physical activity levels after surgery?**
☐ Yes, significantly.
☐ Yes, slightly.
☐ No, my activity levels remain the same.
☐ No, my activity levels have decreased.

**54. What types of sports or physical activities have you found doable after the surgery?**
(Check all that apply)
☐ Walking
☐ Cycling
☐ Swimming
☐ Yoga or Pilates
☐ Strength training
☐ Team sports (e.g., soccer, basketball)
☐ Other: __________
☐ None, I am not able to engage in physical activity.

**55. Have you participated in any type of physiotherapy or rehabilitation program after surgery?**
☐ Yes, and it had a positive impact.
☐ Yes, but it did not have a noticeable impact.
☐ No, but I would like to try physiotherapy or rehabilitation.
☐ No, I do not think physiotherapy or rehabilitation is necessary.

**Medications, Treatments, and Postoperative Diagnostics**

**56. Which best describes your current headache type after CSF leak surgery?**

☐ Rebound intracranial hypertension (high-pressure headache)
☐ Orthostatic headache (worse when standing, better when lying down)
☐ Persistent headache without orthostatic features or high-pressure characteristics

**57. Have you taken Acetazolamide (e.g., Diamox) after surgery?**

☐ Yes
☐ No

If yes, how effective was it?
☐ No improvement
☐ Slight improvement
☐ Significant improvement
☐ Complete resolution
☐ Not applicable

**58. Have you taken Gabapentin or Pregabalin after surgery?**

☐ Yes
☐ No

If yes, how effective was it?
☐ No improvement
☐ Slight improvement
☐ Significant improvement
☐ Complete resolution
☐ Not applicable

**59. Have you taken Theophylline after surgery?**

☐ Yes
☐ No

If yes, how effective was it?
☐ No improvement
☐ Slight improvement
☐ Significant improvement
☐ Complete resolution
☐ Not applicable

**60. Have you taken tricyclic antidepressants after surgery?**

☐ Yes
☐ No

If yes, how effective were they?
☐ No improvement
☐ Slight improvement
☐ Significant improvement
☐ Complete resolution
☐ Not applicable

**61. Have you used pain relievers (e.g., NSAIDs, paracetamol) after surgery?**

☐ Yes
☐ No

If yes, how effective were they?
☐ No improvement
☐ Slight improvement
☐ Significant improvement
☐ Complete resolution
☐ Not applicable

**62. Have you received corticosteroids after surgery?**

☐ Yes
☐ No

If yes, how effective were they?
☐ No improvement
☐ Slight improvement
☐ Significant improvement
☐ Complete resolution
☐ Not applicable

**63. Have you taken beta blockers after surgery?**

☐ Yes
☐ No

If yes, how effective were they?
☐ No improvement
☐ Slight improvement
☐ Significant improvement
☐ Complete resolution
☐ Not applicable

**64. Did you receive a post-surgical epidural blood or fibrin patch?**

☐ Yes
☐ No

If yes, how effective was it?
☐ No improvement
☐ Slight improvement
☐ Significant improvement
☐ Complete resolution
☐ Not applicable

**65. Did you receive an occipital nerve block?**

☐ Yes
☐ No

If yes, how effective was it?
☐ No improvement
☐ Slight improvement
☐ Significant improvement
☐ Complete resolution
☐ Not applicable

**66. Did you undergo a therapeutic lumbar puncture for intracranial pressure management?**

☐ Yes
☐ No

If yes, how effective was it?
☐ No improvement
☐ Slight improvement
☐ Significant improvement
☐ Complete resolution
☐ Not applicable

**67. Did you receive a ventriculoperitoneal (VP) shunt?**

☐ Yes
☐ No

If yes, how effective was it?
☐ No improvement
☐ Slight improvement
☐ Significant improvement
☐ Complete resolution
☐ Not applicable

**68. Have you used GLP-1 receptor agonists (e.g., semaglutide)?**

☐ Yes
☐ No

If yes, how effective were they?
☐ No improvement
☐ Slight improvement
☐ Significant improvement
☐ Complete resolution
☐ Not applicable

**POSTOPERATIVE DIAGNOSTIC TESTS**

**69. Which diagnostic tests were performed after your CSF leak surgery?**

(Check all that apply)
☐ MRI (brain and/or spine)
☐ CT myelography
☐ Dynamic subtraction myelography (DSM)
☐ Ultrasound of optic nerve sheath
☐ Post-operative epidural blood patch
☐ Other: __________

**70. Were any of the following imaging findings reported after surgery?**

(Check all that apply)
☐ Epidural fluid collections
☐ Meningeal enhancement
☐ Brain or cerebellar sagging
☐ Enlarged subarachnoid space
☐ Abnormal venous drainage
☐ None
☐ I don’t know

**71. Headache Impact Test–6 (HIT-6)^1^**

**In the past 4 weeks, how often have you experienced the following?**

| **Question** | **Never** | **Rarely** | **Sometimes** | **Very often** | **Always** |
| --- | --- | --- | --- | --- | --- |
| 1. When you have headaches, how often is the pain severe? | ☐ | ☐ | ☐ | ☐ | ☐ |
| 2. How often do headaches limit your ability to do usual daily activities including household work, work, school, or social activities? | ☐ | ☐ | ☐ | ☐ | ☐ |
| 3. When you have a headache, how often do you wish you could lie down? | ☐ | ☐ | ☐ | ☐ | ☐ |
| 4. In the past 4 weeks, how often have you felt too tired to do work or daily activities because of your headaches? | ☐ | ☐ | ☐ | ☐ | ☐ |
| 5. In the past 4 weeks, how often have you felt fed up or irritated because of your headaches? | ☐ | ☐ | ☐ | ☐ | ☐ |
| 6. In the past 4 weeks, how often did headaches limit your ability to concentrate on work or daily activities? | ☐ | ☐ | ☐ | ☐ | ☐ |

**Reference:**

1. Kosinski M, Bayliss M, Bjorner J, et al. A six-item short-form survey for measuring headache impact: The HIT-6™. *Quality of life research*. 2003;12:963-974.
